# Whole-body magnetic resonance imaging of Li-Fraumeni syndrome patients: observations from a two rounds screening of Brazilian patients

**DOI:** 10.1186/s40644-018-0162-8

**Published:** 2018-08-14

**Authors:** Daniele Paixão, Marcos Duarte Guimarães, Kelvin César de Andrade, Amanda França Nóbrega, Rubens Chojniak, Maria Isabel Achatz

**Affiliations:** 1 Department of Oncogenetics, A.C. Camargo Cancer Center, Professor Antonio Prudente Street, 211 – Liberdade, São Paulo, SP 01509-900 Brazil; 2 Department of Imaging, A.C. Camargo Cancer Center, São Paulo, SP Brazil; 3 Centro de Oncologia, Hospital Sírio-Libanês, São Paulo, Brazil; 4 International Research Center, A.C. Camargo Cancer Center, São Paulo, SP Brazil

**Keywords:** Li-Fraumeni syndrome, Whole-body MRI, Cancer screening, *TP53*, p.R337H mutation

## Abstract

**Background:**

Li-Fraumeni syndrome (LFS) is an autosomal dominant disease that is associated with germline *TP53* mutations and it predisposes affected individuals to a high risk of developing multiple tumors. In Brazil, LFS is characterized by a different pattern of *TP53* variants, with the founder *TP53* p.R337H mutation being predominant. The adoption of screening strategies to diagnose LFS in its early stages is a major challenge due to the diverse spectrum of tumors that LFS patients can develop. The purpose of this study was to evaluate two rounds of whole-body magnetic resonance imaging (WB-MRI) which were conducted as a screening strategy for LFS patients.

**Methods:**

Over a 4-year period, 59 LFS patients underwent two rounds of WB-MRI. Each MRI was characterized as positive or negative, and positive cases were further investigated to establish a diagnosis. The parameters used to evaluate the WB-MRI results included: positive rate, number of invasive investigations of positive results, and cancer detection rate.

**Results:**

A total of 118 WB-MRI scans were performed. Positive results were associated with 11 patients (9.3%). Seven of these patients (11.8%) were identified in the first round of screening and 4 patients (6.7%) were identified in the second round of screening. Biopsies were performed in three cases (2.5%), two (3.4%) after the first round of screening and one (1.7%) after the second round of screening. The histopathological results confirmed a diagnosis of cancer for all three cases. There was no indication of unnecessary invasive procedures.

**Conclusions:**

WB-MRI screening of LFS carriers diagnosed cancers in their early stages. When needed, positive results were further examined with non-invasive imaging techniques. False positive results were less frequent after the first round of WB-MRI screening.

**Electronic supplementary material:**

The online version of this article (10.1186/s40644-018-0162-8) contains supplementary material, which is available to authorized users.

## Background

Li-Fraumeni syndrome (LFS) is a hereditary cancer predisposition syndrome that is associated with germline *TP53* mutations [1]. Those affected by this syndrome are at high risk of developing multiple tumors, both as children and as young adults. The spectrum of tumors that characterize LFS include premenopausal breast cancer, soft tissue sarcoma, osteosarcoma, adrenocortical carcinoma, and brain tumors [2–5]. However, other tumors associated with LFS have been reported as well, including leukemia, Wilms’ tumor, lung, stomach, colorectal, pancreatic, prostate, and choroid plexus carcinomas [6–9].

In Brazil, there is a high prevalence of LFS due to a founder effect [10]. A germline arginine-to-histidine substitution at codon 337 (NC_000017.9: c.1010G > A; p.R337H) is present in 0.3% in the South/Southeastern Brazilian regions. It is estimated that penetrance of LFS associated with the p.R337H mutation is lower than that of other germline *TP53* mutations, with a cumulative lifetime risk of 50 to 60% [10–13].

Effective screening strategies for LFS patients represent a major challenge due to the wide spectrum of tumors and their variable ages at onset that characterize this syndrome. Previous studies have evaluated the use of ^18^F-fluorodeoxyglucose positron emission tomography/computed tomography (^18^F-FDG-PET/CT) as a screening tool for LFS patients [14, 15]. However, since impaired p53 function may enhance the risk of radiation-induced primary tumors in these subjects, the use of this and other radiation-based imaging modalities is not recommended [16–18].

Whole-body magnetic resonance imaging (WB-MRI) is widely used in oncology and it has been proposed as a surveillance strategy for LFS patients [19–24]. This imaging method involves the acquisition of images of the entire body in one or more planes by using fast sequences, and high sensitivity and specificity have been reported for this method in the detection of a wide variety of malignant tumors [20–24]. For example, in a study by Villani et al. [19], a tumor surveillance protocol for both adult and children affected by LFS was proposed which included the use of WB-MRI for cancer screening. A total of 18 LFS patients carrying a *TP53* mutation were monitored for six years. Five malignant tumors were diagnosed in 7 out of 18 patients, including two choroid plexus carcinomas, two adrenocortical carcinomas, and one sarcoma. All of the tumors were diagnosed in asymptomatic patients and a 100% survival rate was reported at the end of the study. In comparison, the survival rate for patients who did not undergo surveillance during the same time period was 23%. These significant results led the National Comprehensive Cancer Network to propose that WB-MRI be included in its guidelines for the management and risk reduction of cancer in children and adults who harbor a *TP53* germline mutation [25].

In another study of WB-MRI conducted by Anupindi et al. [26], 24 children with cancer predisposition conditions, including LFS, paraganglioma-pheochomocytoma syndrome, and rhabdoid tumor syndrome, underwent a total of 50 WB-MRI screenings over five years. Abnormal findings were detected in 18% of the patients examined. Among these findings, a papillary thyroid carcinoma was confirmed to carry a *TP53* mutation. Thus, in this study, WB-MRI had 100% sensitivity and 94% specificity [26].

In 2017, Ballinger et al. [27] conducted a meta-analysis to estimate the performance and frequency of cancer detection by WB-MRI. A total of 578 patients with deleterious germline *TP53* mutations from 13 participating cohorts in six countries were coordinated through the Li-Fraumeni Exploration Research Consortium. Baseline WB-MRI screenings detected 225 lesions in 173 patients. Forty-two malignant neoplasms were identified in 39 individuals, and most of the them were early-stage neoplasms. The resulting cancer detection rate was 7%, although the false-positive rate was 43%. The false-negative rate was not estimated [27].

In 2017, Mai et al. [28] also evaluated a screening protocol that included WB-MRI for examining 116 patients of a National Cancer Institute (NCI) Li-Fraumeni cohort. In this baseline screening, cancers were detected in 6.9% of the cohort, and 34.5% of these lesions required further investigation. The false-positive rate was 29.6%.

Asdahl et al. [29] has cautioned against false positive results and cancer overdiagnosis in LFS screenings which may lead to psychological distress and unnecessary invasive procedures. However, for individuals with LFS, there is sufficient evidence to indicate that the benefits of screening outweighs its risks.

Considering this unique population that has a high frequency of LFS patients, the aim of this study was to evaluate the use of WB-MRI for early tumor detection in Brazilian LFS patients. Two rounds of WB-MRI screenings were performed and various radiological parameters were examined to evaluate this screening approach.

## Methods

### Patients

Between January 2013 and August 2017, LFS patients were recruited for this study from those undergoing follow-up in the Department of Oncogenetics at the A.C. Camargo Cancer Center (São Paulo, Brazil). It was confirmed that the enrolled patients carried a pathogenic germline mutation in the *TP53* gene. A subset of the enrolled patients had a previous history of cancer, yet none of the enrolled patients had evidence of current disease. Patients with a current malignancy or metastatic cancer, pregnant or lactating patients, patients who had surgery or received chemotherapy within the previous four months, and patients who refused to participate in the study were excluded.

### Ethical issues

All patients signed a written informed consent after completing a consultation with a genetic counselor. This study was approved by the local Ethics Committee of the A.C. Camargo Cancer Center (protocol number 1832/13).

### WB-MRI

WB-MRI was performed for 59 LFS patients over a 55-month period in the Nuclear Medicine Division of the Imaging Department, A.C. Camargo Cancer Center. Image acquisition included spin echo and turbo spin echo techniques with coverage tip to toe. Coronal T1-weighted images, short TI inversion recovery (STIR) images, and axial diffusion-weighted images were collected. A 1.5 T MRI instrument (Signa Excite HD; GE Healthcare, Milwaukee, WI, USA) with a quadrature body coil and maximum power gradient of 33 mT/m and a pulse rate of 160 mT/m/s was used. Paramagnetic contrast reagent was not administered in any of the cases. The WB-MRI scanning time was 25–35 min. All images were interpreted by experienced radiologists using an Advantage Windows version 4.2–07 workstation (GE Healthcare). Functool (GE Healthcare) was used to analyze diffusion sequences.

The WB-MRI results were characterized as: (1) positive: defined as suspicious findings of malignancy or undetermined lesions present; or (2) negative: defined as probable benign lesions or an absence of lesions. Patients with positive WB-MRI results were referred to a specialist for clinical management and further follow-up imaging. We evaluated if there was indication of unnecessary invasive procedures in patients with positive results, i.e., biopsy or surgery performed with unconfirmed histopathological result of cancer.

### Radiological parameters

The following radiological parameters were used to evaluate and interpret the WB-MRI results: (1) success rate was measured based on the number of exams performed without complication and without sedation/anesthesia; (2) positive rate was measured according to the number of positive WB-MRI results; (3) recall rate was measured based on the number of patients requiring further investigation after WB-MRI; and (4) cancer detection rate was measured based on the number of malignancies diagnosed by WB-MRI.

## Results

A total of 118 WB-MRI scans were performed for 59 LFS patients (35 females, 24 males) from 23 families with a mean age of 38 years (range: 2–71) at the time of their initial WB-MRI examination. Among these patients, 50 (85%) carried the founder mutation, p.R337H, and 9 (15%) carried other germline *TP53* mutations. A total of 27 (45%) patients had a prior history of cancer, with 12 previously receiving a diagnosis of multiple primary tumors (Additional file 1: Table S1). A second WB-MRI was performed for all of the patients after a minimum interval of 12 months.

The initial round of WB-MRI had a low positivity rate (11.8%), a low recall rate (11.8%), few invasive investigations (3.4%), and a cancer detection rate of 3.4% (Table 1). The success rate for the execution of the initial WB-MRI screenings was high (95%). Only two pediatric patients and one adult patient who was claustrophobic required sedation. Positive results were obtained for seven patients, and further investigations with other imaging exams were performed to rule out suspected malignant lesions (Table 2). Two of these patients required biopsy and the histopathologic findings confirmed a diagnosis of cancer in both patients.

**Table 1:** Summary of WB-MRI results according to various radiological parameters

Radiological parameters	1^st^ WB-MRI, *n* = 59 % (n)	2^st^ WB-MRI, *n* = 59 % (n)	Total, *n* = 118 % (n)
Positivity rate	11.8 (7)	6.7 (4)	9.3 (11)
Recall rate	11.8 (7)	6.7 (4)	9.3 (11)
Cancer detection rate	3.4 (2)	1.7 (1)	2.5 (3)
Success rate	95.0 (56)	100.0 (59)	98.0 (116)

**Table 2:** Further investigation of the positive cases identified in the first and second-round of WB-MRI screenings

Patient ID no.	M/F	Age (y)	*TP53* mutation	WB-MRI findings	Further exams	Imaging diagnosis	Invasive exams	Cancer diagnosis
Y0012T044	F	40	p.R337H	Hyposignal area on T1, high signal focuses on STIR in left humeral head, nonspecific aspect.	Shoulder MRI	Vascular ectasia	No	No
Y0012T049	F	61	p.R337H	Rounded image in distal metaphyseal region of the right femur (2.2 cm) with hyposignal on T1 and hypersignal on STIR.	Leg MRI	Lobulated image in right femur compatible with enchondroma	No	No
Y0079T016	F	41	p.T125T	Sacral nodule (20 mm), nonspecific,	Lumbosacral spine CT	Enostoses	No	No
Y0099T001	F	34	p.R337H	Nodular area (17 mm) in the retroperitoneum, adjacent to pancreatic cephalic region.	Abdominal MRI	Unilocular pancreatic cyst	No	No
Y0102T000	F	17	p.R337H	Oval images in renal cortical, 16 mm in the lower pole of the right kidney and 20 mm in the upper pole of the left kidney.	Abdominal/ Pelvis MRI	Nodule with irregular borders and heterogeneous signal, predominantly high in T1 and intermediate in T2, with restrictions on diffusion sequence. Approximately, 23 × 18 × 18 mm^3^ in right kidney	Surgical resection	Renal cell carcinoma
Y0183T001	M	19	p.T125T	Image on the left lenticular nucleus, 8 mm.	Brain MRI	Cystic image in the left putamen, compatible with Virchow-Robin space	No	No
Y0352T000	F	29	p.R306X	Nodule 35 × 28 mm^2^ at the left sacroiliac joint.	Pelvis MRI	Solid mass lesion 43 × 34 × 30 mm^3^, located in the posterior-inferior region of the left sacroiliac joint, with bone cortical irregularity in the sacral margin	Surgical resection	Grade 1 chondrosarcoma
Y0102T000	F	18	p.R337H	Nodule in the right adrenal gland with hypointense signal on T1, hyperintense signal on STIR, and diffusion sequence. Measures 15 mm, indeterminate aspect.	Abdominal MRI	Nodule in the right adrenal gland, non-aggressive features	No	No
Y0015T011	M	34	p.R337H	Images in humeral heads with hypointense signal on T1 and hyperintense signal on STIR, measuring up to 9 mm, suggestive of subchondral cysts.	Shoulder MRI, CT and scintigraphy	Radiolucent oval image in the scapular region with non-aggressive features, slight increase in bone metabolism, no change in blood flow, suggestive of cystic lesion or enchondroma	No	No
Y0012T012	M	61	p. R337H	Expansive lesion in the right humerus, measuring 48 × 34 mm^2^, with isosignal to muscle on T1, hyperintense signal on STIR, and restriction on diffusion sequence.	Shoulder MRI	Expansive lesion in the right axillary region, next to the distal roots of the brachial plexus, measuring 32 × 22 mm^2^, with hypointense signal on T1, hyperintense signal on STIR, suggestive of malignancy of neural sheath or soft tissue sarcoma	Biopsy (axilar lymph node)	High-grade sarcoma with muscle differen- tiation
Y0171T005	M	47	p. R337H	Subcutaneous nodule on the left shoulder.	Shoulder ultrasound	Echogenic nodule in the subcutaneous tissue of the left shoulder, measuring 65 × 57 × 12 mm^3^, characteristics of lipoma	No	No

**Fig. 1:**
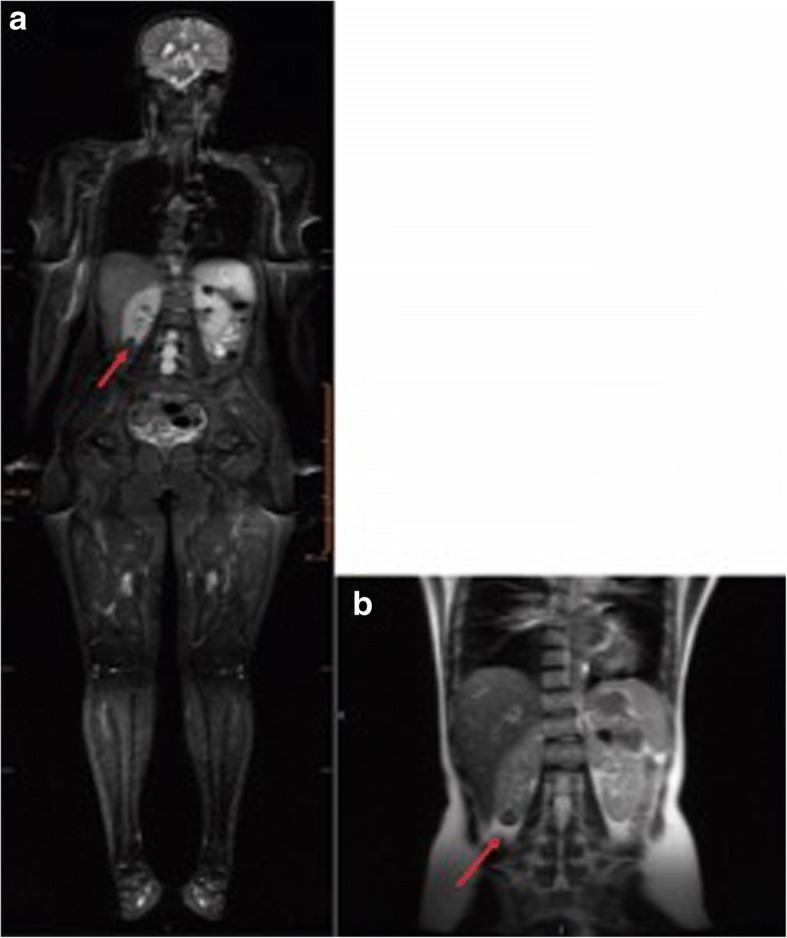
Detection of a renal lesion by WB-MRI in LFS patient, Y0102T000. **a** STIR image of a 19-year-old with cortical oval kidney lesions in the right lower pole (16 mm) and in the left upper pole (20 mm) (indicated with arrows). **b** An abdominal MRI detected a complex nodule in the lower third of the right kidney, and it measured 23 × 18 × 18 mm^3^ (indicated with an arrow)

**Fig. 2:**
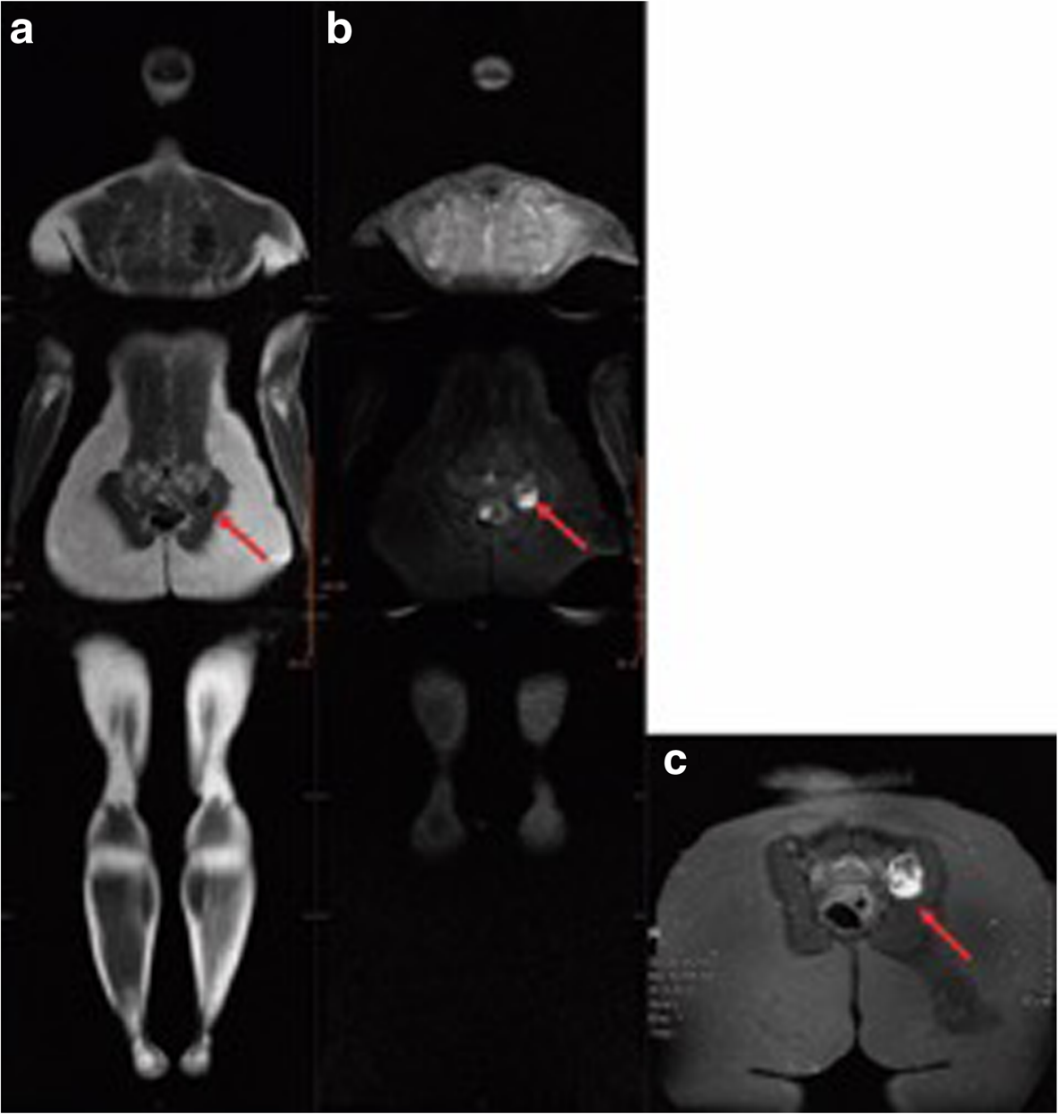
Detection of a sacroiliac joint lesion by WB-MRI and pelvic MRI images of LFS patient, Y0352T000. A T1-weighted image (**a**) and a STIR image (**b**) were obtained with WB-MRI. **c** MRI of the sacroiliac region detected a solid expansive lesion in the posterior-inferior region of the left sacroiliac joint. Bone cortical irregularity was observed in the sacral margin and it was accompanied by bone edema (indicated with arrow)

One of the positive patients was a 19-year-old female carrier of p.R337H (Y0102T000) with a previous history of multiple primary tumors. Her initial WB-MRI detected bilateral renal cortical alterations. An abdominal MRI further detected an enhanced solid lesion in the right kidney (Fig. 1a and b). Pathological findings confirmed papillary renal cell carcinoma (T1AN0M0, stage I). The second positive patient was a 29-year-old female carrier of p.R306X (Y0352T000). She was asymptomatic and had no previous history of cancer. Her initial WB-MRI screening detected a nodule in the left sacroiliac joint (Fig. 2a and b). A pelvic MRI further showed an enhanced solid expansive lesion present (Fig. 2c). Pathological findings confirmed the lesion to be grade 1 chondrosarcoma.

The positive rate and recall rate for the second round of WB-MRI screenings for all 59 patients were both 6.7%. The success rate was 100% and sedation/anesthesia was not needed for any of the screenings (Table 1). Four positive results were detected and further examinations were performed (Table 2). A biopsy was only performed for one patient and the histopathological results confirmed cancer. Meanwhile, the remaining three patients had their suspected lesions ruled as benign. Overall, the cancer detection rate for the second round of WB-MRI examinations was 1.7%, which is lower than the cancer rate in the first round of WB-MRI screenings. In addition, there was a lower indication of further investigation in the second round of WB-MRI examinations.

**Fig. 3:**
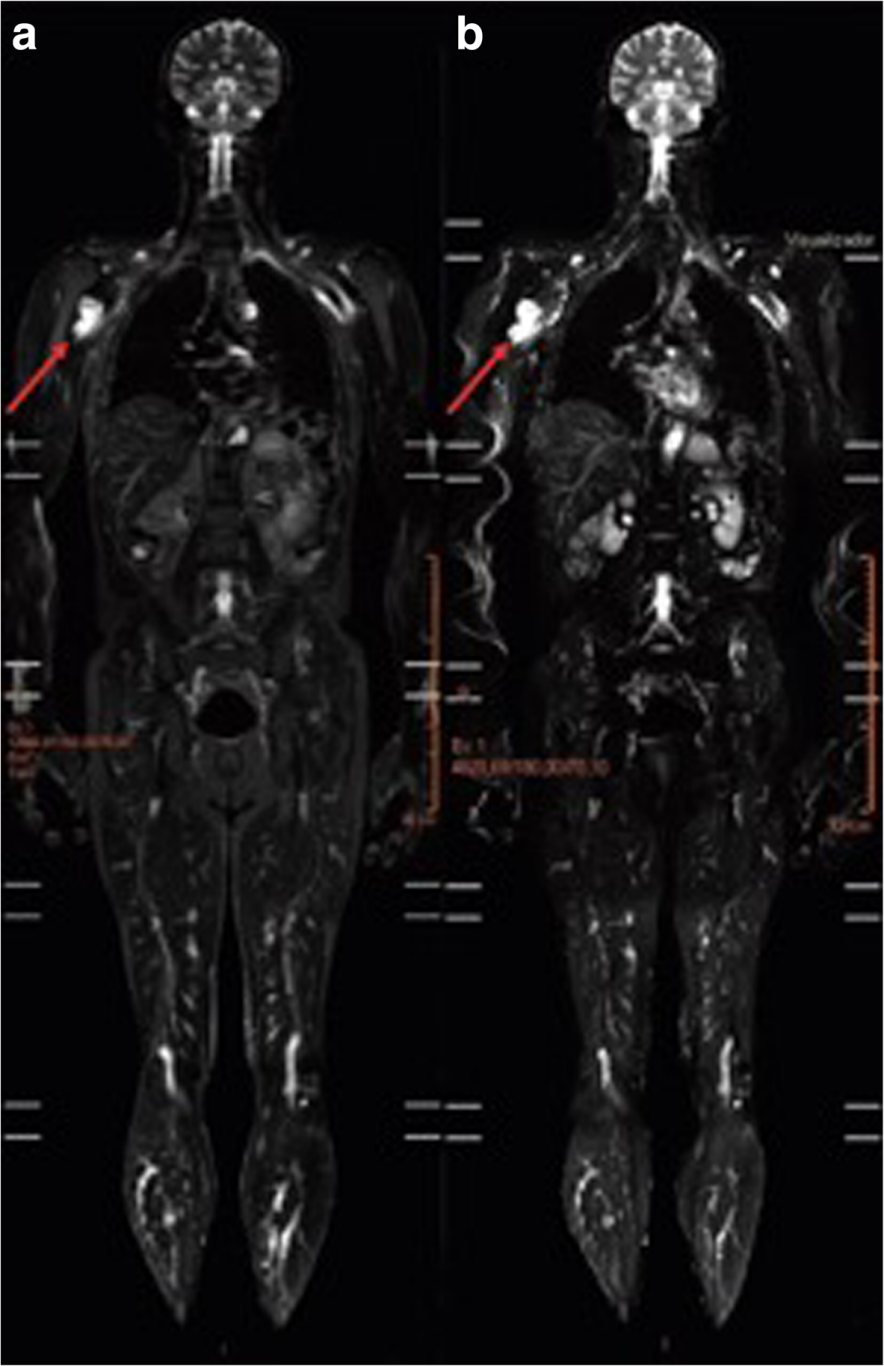
WB-MRI of LFS patient, Y0012T012. A STIR image (**a**) and diffusion-weighted imaging (**b**) from WB-MRI. An expansive lesion (48 × 34 mm^2^) in contact with the short head of the biceps was detected in the proximal segment of the right humerus. The lesion exhibited a hyperintense signal on STIR and restriction on diffusion-weighted imaging scans (indicated with arrows)

The confirmed positive finding in the second round of WB-MRI screenings involved a 61-year-old asymptomatic male carrier of p.R337H (Y0012T012) with a previous history of a thyroid cancer at the age of 54. An expansive lesion was detected in the proximal segment of the right humerus that was in contact with the short head of the biceps (Fig. 3a and b). This finding suggested a malignancy of the neural sheath or a soft tissue sarcoma. Pathology subsequently confirmed the presence of a high-grade sarcoma with muscle differentiation.

## Discussion

The adoption of a screening strategy for the early detection of tumors in LFS patients remains a challenge due to the wide spectrum of tumors that can develop. However, recent studies have shown that WB-MRI can be effective for the screening of patients with cancer predisposition syndromes, including LFS [19, 26].

WB-MRI with diffusion-weighted imaging has the advantage that it is a noninvasive technique that does not require exposure to ionizing radiation or paramagnetic contrast reagent. This is particularly relevant for LFS patients who are advised to avoid exposure to ionizing radiation due to their higher risk of developing radiation-induced primary tumors [14, 16–18]. In the present study, 118 WB-MRI screenings were performed for 59 carriers of germline *TP53* mutations. After two rounds of WB-MRI screenings, cancers were detected in two (4%) out of 50 carriers of the founder mutation, p.R337H, and one cancer (11%) was detected among 9 carriers of the other germline *TP53* mutations. For the three positive cases, biopsies were necessary and the histopathological results confirmed malignant lesions in each case: renal cell carcinoma, grade 1 chondrosarcoma, and soft tissue sarcoma. There was no indication of unnecessary invasive procedures. All of the lesions were also detected in their early stages of development in asymptomatic individuals. Consequently, surgical resection was the primary treatment with no additional need for chemotherapy or radiation treatment. Thus, WB-MRI screening in the present cohort was characterized by a high success rate, minimal need for sedation, and good tolerance and acceptance of the screening protocol.

It is important to note that one of the three diagnosed cancers were detected during the second round of WB-MRI screenings that were performed 12 months after the initial WB-MRI screenings. This result supports the relevance of annual examinations for LFS patients. These results are also consistent with previous observations that WB-MRI facilitates the early detection of malignant tumors [19, 26–28]. Low positivity and recall rates were observed, and further investigations of positive results predominantly involved radiological methods. Only a small proportion of the patients examined underwent an invasive exam with biopsies performed to confirm malignant tumors.

High false-positive rates in screening protocols have been shown to increase the potential for excessive subsequent investigations and unnecessary invasive procedures [29]. In the present study, a significantly lower positive rate was observed in the second round of WB-MRI screenings, thereby reducing the need for further investigations. This result was expected for a screening process and these data reinforce the findings of Ballinger et al. [27] that WB-MRI may be an integral part of a screening strategy for this high-risk population of patients.

It should be noted that there were limitations associated with the present study. For example, there was a high proportion of younger patients (< 40 y) included in the cohort examined, and the majority of the carriers examined harbored the p.R337H mutation. The latter has been characterized as having a reduced penetrance at younger ages compared to other classic mutations associated with LFS patients [10–13]. Among the 50 patients who carried the p.R337H mutation, the cancer detection rate was low. For example, it has been reported that approximately 15% of individuals carrying p.R337H develop cancer by the age of 30, whereas patients who carry other mutations in the *TP53* gene present a risk of 50% up to the age of 35 years [5, 10, 30]. These observations may account for the low rate of cancer detection that was observed in the present study.

Another limitation of the present study was that the WB-MRI results were not compared with other imaging modalities. However, to date, there is no gold standard that has been established for cancer screenings of LFS patients. Thus, it is not possible to evaluate the effectiveness of the exams performed.

A major challenge in the screening of LFS patients is the management of pediatric cases. A protocol proposed by Villani et al. [19] indicates specific tests for children with LFS, particularly for the detection of adrenocortical carcinomas, brain tumors, and hematological cancers, and the protocol recommends performing WB-MRI annually to detect sarcomas. There is still no consensus regarding the best age at which to start WB-MRI screenings, and its application to young children is challenging due to the need for sedation or general anesthesia, neither of which are without risks. Anupindi et al. [26] reported that 58% of pediatric patients required sedation for WB-MRI screenings, and complications from the procedure were documented in six patients. In the present study, use of sedation was necessary in two pediatric cases involving a 2-year-old and a 3-year-old, and no further complications were observed. Thus, sedation for WB-MRI has generally been observed to be a safe procedure. However, the risk-benefit of WB-MRI with sedation/general anesthesia for children remains to be confirmed with further studies.

## Conclusion

Despite the limitations associated with this study, a large cohort of LFS patients were evaluated with two rounds of WB-MRI screenings, including a large proportion of Brazilian LFS patients carrying the p.R337H mutation. Our results indicate that cancer screening based on WB-MRI facilitated the early detection of malignant neoplasms and it was characterized by lower recall rates and fewer follow-up invasive investigations. Furthermore, in the second round of screening, fewer positive results were observed. Therefore, we recommend that WB-MRI should be performed as a complementary method to other proposed tests for the surveillance of LFS patients, although longitudinal studies are still needed to better evaluate the effectiveness and long-term impact of WB-MRI on the survival of LFS patients.

## Additional file

Additional file 1: **Table S1.** Characteristics of the LFS patients screened in this study. Clinical details and germline *TP53* mutations of the LFS patients examined. Reference sequences used for annotating TP53 mutations included: NC_000017.9 (NCBI36/hg18, Chr17:7512445..7531642), NM_000546.4, and UniProt P04637 from GenBank). Mutations are presented according to HGVS nomenclature. Abbreviations: F, female; M, male; ADR, adrenocortical carcinoma; STS, soft tissue sarcoma; CNS, central nervous system. (DOCX 15 kb)

## Abbreviations

18F-FDG-PET/CT: 18F-fluorodeoxyglucose positron emission tomography/computed tomography
LFS: Li-Fraumeni syndrome
NCI: National Cancer Institute
STIR: Short TI inversion recovery
WB-MRI: Whole-body magnetic resonance imaging
