# Supplementary material for: Whole-body magnetic resonance imaging of Li-Fraumeni syndrome patients: observations from a two rounds screening of Brazilian patients
Source: Cancer Imaging. 2018 Aug 14;18:27. doi: 10.1186/s40644-018-0162-8 (PMC6092833; doi:10.1186/s40644-018-0162-8)
Supplement: Supplementary file 1 — Table S1. Characteristics of the LFS patients screened in this study. Clinical details and germline TP53 mutations of the LFS patients examined. Reference sequences used for annotating TP53 mutations included: NC_000017.9 (NCBI36/hg18, Chr17:7512445..7531642), NM_000546.4, and UniProt P04637 from GenBank). Mutations are presented according to HGVS nomenclature. Abbreviations: F, female; M, male; ADR, adrenocortical carcinoma; STS, soft tissue sarcoma; CNS, central nervous system. (DOCX 15 kb) [file 40644_2018_162_MOESM1_ESM.docx]

**SUPPLEMENTARY MATERIALS**

**Table S1**

| **Identification**  **no.** | **Family** | ***Type of TP53***  **mutation** | **Gender** | **Current Age (y)** | **Previous history of cancer (age at cancer diagnosis, y)** |
| --- | --- | --- | --- | --- | --- |
| Y0012T007 | Y0012 | p.R337H | F | 36 | No |
| Y0012T011 | Y0012 | p.R337H | M | 37 | Thyroid (32) |
| Y0012T012 | Y0012 | p.R337H | M | 61 | Thyroid (54) |
| Y0012T022 | Y0012 | p.R337H | M | 68 | No |
| Y0012T023 | Y0012 | p.R337H | F | 30 | No |
| Y0012T025 | Y0012 | p.R337H | F | 49 | No |
| Y0012T026 | Y0012 | p.R337H | M | 27 | No |
| Y0012T027 | Y0012 | p.R337H | M | 67 | Prostate (62) |
| Y0012T029 | Y0012 | p.R337H | F | 52 | No |
| Y0012T044 | Y0012 | p.R337H | F | 43 | No |
| Y0012T049 | Y0012 | p.R337H | F | 62 | No |
| Y0012T055 | Y0012 | p.R337H | F | 70 | Breast (58;65) |
| Y0012T076 | Y0012 | p.R337H | M | 45 | No |
| Y0012T082 | Y0012 | p.R337H | M | 25 | No |
| Y0012T084 | Y0012 | p.R337H | M | 36 | No |
| Y0012T085 | Y0012 | p.R337H | M | 37 | No |
| Y0012T089 | Y0012 | p.R337H | F | 33 | Breast (27;27) |
| Y0012T090 | Y0012 | p.R337H | M | 37 | No |
| Y0012T095 | Y0012 | p.R337H | M | 17 | No |
| Y0015T000 | Y0015 | p.R337H | F | 34 | ADR (7), Kidney (8) |
| Y0015T001 | Y0015 | p.R337H | M | 73 | Thyroid (70) |
| Y0015T003 | Y0015 | p.R337H | F | 29 | Thyroid (26) |
| Y0015T004 | Y0015 | p.R337H | F | 29 | Thyroid (24), Pheochromocytoma (25) |
| Y0015T010 | Y0015 | p.R337H | F | 61 | No |
| Y0015T011 | Y0015 | p.R337H | M | 35 | No |
| Y0015T029 | Y0015 | p.R337H | F | 32 | No |
| Y0027T004 | Y0027 | p.R337H | M | 40 | No |
| Y0099T000 | Y0099 | p.R337H | F | 41 | Breast (32) |
| Y0099T001 | Y0099 | p.R337H | F | 34 | No |
| Y0102T000 | Y0102 | p.R337H | F | 19 | Lymphoma (7),  STS (10;10;10;12;14;14;14;16), ADR (10) |
| Y0102T001 | Y0102 | p.R337H | M | 24 | CNS (19) |
| Y0131T001 | Y0131 | p.R337H | F | 56 | Breast (43), Thyroid (46) |
| Y0131T002 | Y0131 | p.R337H | F | 10 | No |
| Y0154T000 | Y0154 | p.R337H | F | 55 | Breast (48) |
| Y0158T001 | Y0158 | p.R337H | F | 54 | No |
| Y0171T000 | Y0171 | p.R337H | F | 20 | ADR (7 months) |
| Y0171T001 | Y0171 | p.R337H | M | 50 | No |
| Y0171T005 | Y0171 | p.R337H | M | 47 | No |
| Y0171T007 | Y0171 | p.R337H | F | 15 | No |
| Y0184T000 | Y0184 | p.R337H | M | 60 | ADR (50), Prostate (50),  STS (60) |
| Y0184T001 | Y0184 | p.R337H | M | 33 | No |
| Y0187T000 | Y0187 | p.R337H | F | 69 | Breast (60; 63),  Lymphoma (60) |
| Y0225T002 | Y0225 | p.R337H | M | 31 | No |
| Y0228T000 | Y0228 | p.R337H | F | 51 | Breast (47), Lung (48),  STS (49) |
| Y0232T000 | Y0232 | p.R337H | F | 57 | Pancreas (45), Breast (48) |
| Y0236T001 | Y0236 | p.R337H | M | 40 | No |
| Y0241T000 | Y0241 | p.R337H | F | 4 | ADR (3) |
| Y0241T002 | Y0241 | p.R337H | F | 25 | No |
| Y0242T001 | Y0242 | p.R337H | F | 49 | No |
| Y0254T000 | Y0254 | p.R337H | F | 33 | Breast (31) |
| Y0065T001 | Y0065 | p.V197M | M | 36 | Skin (non-melanoma) (30) |
| Y0079T005 | Y0079 | p.T125T | F | 67 | No |
| Y0079T016 | Y0079 | p.T125T | F | 43 | Breast (36) |
| Y0097T000 | Y0097 | IVS8+ 1G>A | M | 39 | Rhabdomyosarcoma (1), STS (28;36) |
| Y0097T002 | Y0097 | IVS8+ 1G>A | F | 5 | ADR (6m) |
| Y0183T000 | Y0183 | p.T125T | F | 54 | Breast (44),  Melanoma (47) |
| Y0183T001 | Y0183 | p.T125T | F | 21 | No |
| Y0183T005 | Y0183 | p.T125T | M | 23 | No |
| Y0352T000 | Y0352 | p.R306X | F | 29 | Breast (21) |
